# Assessing the impact of the 2008 health reform in Ecuador on the performance of primary health care services: an interrupted time series analysis

**DOI:** 10.1186/s12939-021-01495-2

**Published:** 2021-07-22

**Authors:** Sergio E Flores Jimenez, Miguel San Sebastián

**Affiliations:** Department of Epidemiology and Global Health, Umeå University, Umeå, Sweden

**Keywords:** Ambulatory care sensitive conditions, interrupted time series analysis, primary health care, health reform, Ecuador

## Abstract

**Background:**

In 2008, Ecuador started a national health reform based on the principles of Alma Ata to achieve Universal Health Coverage. While coverage indicators have increased, a systematic assessment of the impact of the reform on the delivery of health services at primary level is lacking. The aim of this study was to assess the impact of the 2008 health reform on the performance of primary health care services in Ecuador.

**Methods:**

Ambulatory Care Sensitive Conditions (ACSC) are a subset of diseases where hospital admission is potentially avoidable by high quality well-functioning primary care. Thus, observing the behaviour of ACSC hospitalizations can serve as an indicator of how the primary health care level is performing. Crude and adjusted rates, stratified by sex, were calculated from ten selected ACSC hospitalization discharges during 22 years of data representing 11 years before and after the health reform. An interrupted time series analysis was then conducted by applying a negative binomial regression and adjusting for overdispersion and autocorrelation.

**Results:**

Overall higher crude and adjusted rates for ACSC hospitalizations were observed in women compared to men; both increased gradually since the start of the observation, reaching a peak around 2010, and then started a downwards trend. In men, the incidence rate ratio increased significantly by 3 % per year during the period before the intervention. During the first year after intervention, an increase (13 %) was detected, and then a statistically significant 1 % decrease (IRR = 0.99; 95 % CI: 0.98, 0.99) was observed in the ACSC rate ratio per year in the period after the intervention. Similar trends and effect sizes were found for women.

**Conclusions:**

The study revealed significant decreasing trends of the ACSC hospitalization rates in both sexes, indicating an improvement of the performance of the primary health care services following the 2008 national health reform. A continuous strengthening of the primary care model as well as a regular monitoring of ACSC hospitalization rates in the country is recommended. A health economic evaluation considering hospitalizations avoided and associated costs is also advisable.

**Supplementary Information:**

The online version contains supplementary material available at 10.1186/s12939-021-01495-2.

## Background

Many countries throughout the world have recently framed their national health policies towards the goal of achieving Universal Healthcare Coverage (UHC) [1]. This strategy advocates quality health care services accessible to all people according to need, as well as financial protection to avoid users´ economic hardship. In order to achieve UHC, the World Health Organization has suggested the need to raise funds from taxes, to reduce the reliance on direct payments to finance services, and to improve efficiency and equity under a framework that organizes and delivers care that best meets the population needs [2].

Starting in the 2000 s, UHC has been prominent in the political agendas of many Latin American countries based on comprehensive primary health care (PHC) models [3]. All countries in this region have committed to the implementation of UHC through its inclusion in their policy documents. These countries are also making efforts to reform their health financing to increase the pooling of funds, guaranteeing access to an essential package of services and providing financial protection to the most vulnerable [4]. Consequently, several countries have reorganized their health systems to address structural fragmentation, decentralize health system functions to local levels of government, develop robust regulatory functions, and separate purchasing and provider roles [5]. An article from 2015 assessed the progress towards UHC using data from 112 household surveys in 20 Latin American countries between 1990 and 2013 monitoring several indicators of health service coverage (antenatal visits, full immunization for children, breast and cervical cancer screening), treatment coverage (skilled birth attendant, appropriate treatment for children´s diarrhea and respiratory infection) and financial protection (catastrophic out-of-pocket and impoverishing spending on health care services). The review concluded that all countries were moving in the right direction but were still far from achieving full UHC [6].

One of the countries which has undertaken important structural changes in both the constitutional reform of the right to health and the organization of public institutions has been Ecuador [7]. In 2008 the constitution was reformed to make health care a right [8]; it sought to build a new health care model that was not centred around diseases and curative health services, but on people, family, and community, with a strong perspective of health promotion, prevention, and rehabilitation. The model, built on the principles of Alma Ata, started by offering free cost health services to the entire population, which increased the utilization of healthcare services by 300 % during the period between 2008 and 2016. In addition to a strong investment in health infrastructure, the country increased the salaries of the health workforce and recruited more than 5,000 health professionals between 2012 and 2015 [9].

Two studies have assessed the role of the Ecuadorian health reform on decreasing socioeconomic inequalities in health care utilization and health outcomes, respectively [10, 11]. In the first one, a decrease in income inequalities in curative visits was observed after the reform. The second study revealed a more complex picture where most socioeconomic inequalities in skilled birth attendance, but not in cervical cancer screening or the use of modern contraceptives, had decreased during the reform period.

However, except by information related to coverage indicators [9], no study has systematically assessed the impact of the reform on the functioning of health services at the primary level. The aim of this study was to assess the impact of the 2008 health reform on the performance of primary health care services in Ecuador.

## Methods

### Design and data source

Ambulatory Care Sensitive Conditions (ACSC) are a subset of diseases where hospital admission is potentially avoidable by preventing the onset of disease, controlling an acute episodic illness, or managing a chronic condition effectively by providers at primary care level. With high quality well-functioning primary care, the need for hospital care for these conditions should be reduced [12, 13]. Therefore, observational evidence on the behaviour of ACSC hospitalizations can serve as an indirect indicator of how a primary health care system is performing. This approach has been previously used in the Latin American region [14–17].

To evaluate the effectiveness of the 2008 health reform in Ecuador through its impact on the changes of ACSC, a quasi-experimental study design was undertaken using an interrupted time series analysis (ITSA). This design is commonly used to assess the consequences of a variety of policy issues in various fields, such as community interventions, public policy, regulatory actions, environmental policies, financial economics, health technology assessment, and health policies [18]. It is particularly suited to evaluate public health interventions introduced at the population level over a clearly defined period of time [19, 20].

Ten ACSC covering 22 years of data spanning 11 years before and after the health reform were selected. The ACSC included gastroenteritis, diabetes, diarrhoea, angina, heart failure, pneumonia, chronic obstructive pulmonary disease, asthma, cellulitis, and urinary tract infections. They were chosen based on previous studies conducted in Ecuador [14, 15] and retrieved following their respective International Classification of Diseases (ICD-9/10) codes.

The total number of national hospital discharges – including both conditions identified as ACSC and non-ACSC - aggregated by year (1997–2018), sex, and age was extracted from the National Institute of Statistics and Census (INEC in Spanish) database of Ecuador. This information is publicly available at: https://www.ecuadorencifras.gob.ec/camas-y-egresos-hospitalarios/. National populations per age brackets and sex were obtained for every year using the same source. The world standardized population for each age bracket was obtained from the World Health Organization (WHO) [21]. All this data was inserted into an Excel file and then transferred into a Stata database.

### Data analysis

The crude rate per 1000 people was calculated based on the number of ACSC reported per year and the population for each respective age bracket; the age adjusted ACSC rate was calculated through the direct standardization method using the WHO standard population as a reference. The crude and adjusted rates were then plotted into a multiple overlaid connected line graph. Raw data are provided as supplementary material (Table 1S).

Since the outcome was a count, a Poisson regression model was first used including the total population as the denominator to convert the outcome into a rate and adjust for any potential changes in the population over time. The log of the expected outcome was predicted with a linear combination of the predictors:

ln({acsc}) = βo (Intercept) + β1preslope + β2intervention + β3postslope + ε*t*.

**Fig. 1:**
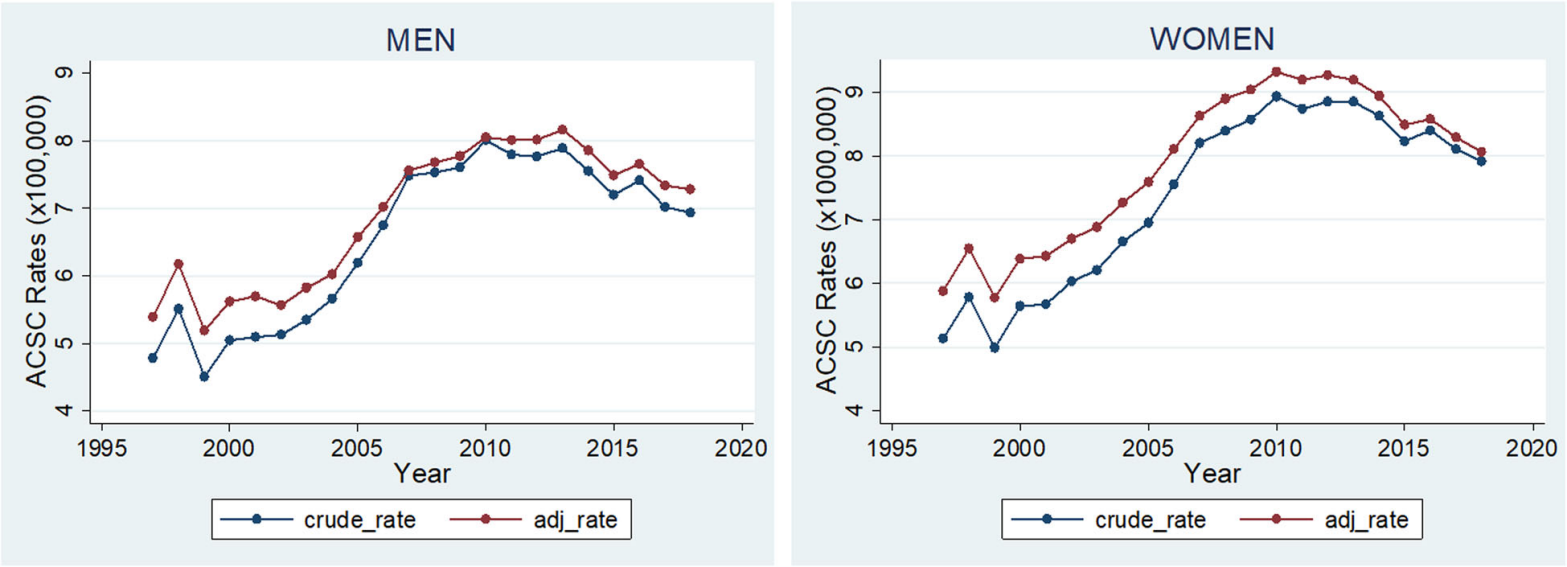
Trends in crude and age-adjusted ambulatory care sensitive conditions ACSC rates (x 100,000) by sex, 1997–2018, Ecuador

Initial analysis suggested a moderate degree of overdispersion, so a more flexible negative binomial model was used for all analyses. Robust standard errors using the Huber-White sandwich estimator to control for mild violations of underlying assumptions (normality, heteroscedasticity, or large residuals) were calculated. Rate ratios (RR) and 95 % confidence intervals (95 % CI) were obtained as measures of association. As a complementary analysis, total hospitalization rates in both sexes over the same period were also calculated (Fig. 1S in the Additional file 1). All statistical analyses were conducted using Stata 16.1.

### Ethical approval

All the data obtained are publicly available online and provided by the government’s national agency of statistics in an aggregated format. No specific ethical procedures had to be undertaken regarding data collection or anonymity.

## Results

In total, 2,245,339 (11.02 % of the total) ACSC hospitalizations were included in the period 1997–2018 (supplementary Table 2S). Overall higher crude and adjusted rates for ACSC hospitalizations were observed in women compared to men, both increasing gradually since the start of the observation, reaching a peak around 2010 and then starting a downwards trend (Fig. 1). In men, the incidence rate ratio increased significantly by 3 % per year during the period before the intervention. During the first year after intervention, an increase (13 %) was detected, followed by a statistically significant 1 % decrease (IRR = 0.99; 95 % CI: 0.98, 0.99) in the ACSC rate ratio per year in the period after the intervention. Similar trends and effect sizes were found for women (Table 1).

**Table 1:** Segmented negative binomial regression analysis including incidence rate ratios (IRR) and their 95 % confidence intervals (95 % CI); pre- and post-intervention by sex, 1997–2018, Ecuador

	Men		Women	
	IRR	(95 % CI)	IRR	(95 % CI)
Pre-intervention slope	1.03	(1.02–1.04)*	1.04	(1.03–1.05)*
Intervention	1.13	(1.05–1.21)*	1.11	(1.05–1.17)*
Post-intervention slope	0.99	(0.98–1.00)	0.99	(0.98–0.99)*
Pre- post trend difference	0.95	(0.95–0.97)*	0.95	(0.94–0.96)*

* *p* < 0.05

**Fig. 2:**
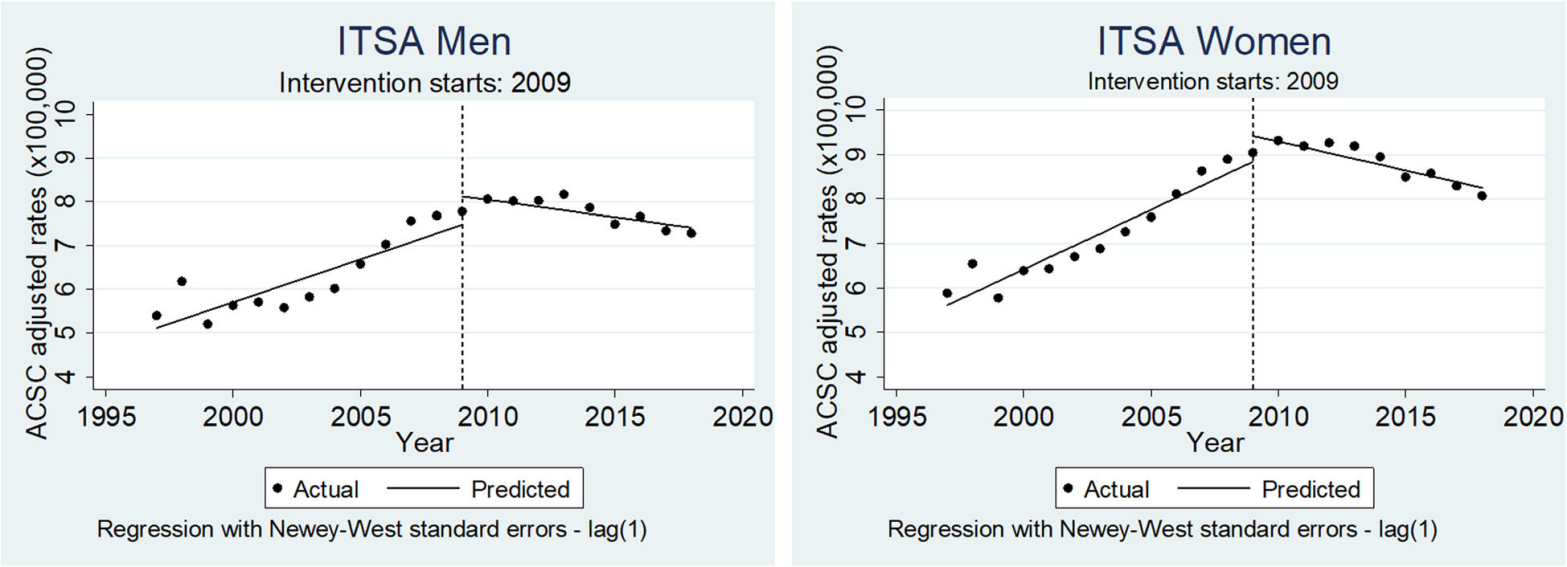
Trends in ambulatory care sensitive conditions (ACSC) age-adjusted rates (per 100,000 population) with fitted regression line before (1996–2008) and after (2009–2018) the health reform in Ecuador in men (left) and women (right); (ITSA=interrupted time series analysis)

When calculating the pre- and post-trend difference, a statistically significant 5 % decrease (IRR = 0.95; 95 % CI: 0.94, 0.96) in the ACSC rate ratio was observed in both sexes (Fig. 2; Table 1).

## Discussion

This study assessed the performance of primary health care services, measured by the ACSC, after the implementation of a national health reform in Ecuador. The findings have revealed that the health reform seemed to have attained a positive effect on improving the performance of the health care system. These results expand a previous study describing ACSC trends in Ecuador in the period 2002–2012, where no intent to relate the trends with the 2008 health reform was performed [14]. Our results are in line with an Inter-American Development Bank report on ACSC in selected countries of the Latin American region using data ranging from 2001 to 2009 (15). While the report found an average percentage of ACSC for all hospitalizations ranging between 10.8 % (in Costa Rica) and 21.6 % (in Colombia), our average percentage for the study period was 15.1 % for men and 10.1 % for women.

The main finding of this study was the significant decrease in the trend of ACSC hospitalization rates for both men and women after the reform started in 2008. The pattern of increasing trends immediately following the intervention may be due to the refractory period between legislation and implementation. A health care reform, from legislative approval to institutional implementation, requires time to be set in place, let alone to start seeing its effects. This is especially relevant in this case considering the national scale and complexity of the intervention.

Despite the decrease in rates of ACSC after the reform, they were still higher than in the 90 s before any reform was implemented. One explanation of this apparently contradictory situation could be that overall hospitalization rates were low in the 1990 s due to a lack of access to these facilities. This was observed when further analyses were conducted that examined total hospitalization rates in both sexes over the same period (see Fig. 1 S in the Appendix). Hospitalization rates were low during the 1990 s and beginning of the 2000 s increasing significantly after the health reform.

The positive impact of the reform on primary health care services is consistent with studies conducted in the region. In the 1990 s, Brazil introduced the Family Health Program (Programa de Salud Familia), which developed a new, more robust model of primary health care services designed to provide accessible, first contact, comprehensive, and whole person-centred care [22]. Different studies carried out in 2008, 2009, and 2011 found that overall hospitalization rates due to ACSC significantly decreased with varying degrees between both sexes [16, 17, 22].

To our knowledge, there has not been conducted a comprehensive evaluation of the health reform in Ecuador. While different stakeholders linked to the reform implementation have advocated its success [9, 23], other scholars have claimed that the reform has been insufficient. Except for the higher budget allocated, limited changes have been implemented in the structure of the health system [24, 25] which could explain the slow decreasing pattern of ACSC post-reform observed.

### Methodological considerations

Several issues should be considered when interpreting these findings. Interrupted time series analysis has been considered as one of the strongest quasi-experimental research designs, particularly when a randomized trial is unfeasible or unethical [18, 19]. The availability of longitudinal population rates spanning eleven years before and after the health reform policy can be considered enough to capture the differences in hospitalization rates and therefore the effects of the intervention. While there is a strong assumption in the design that the reform is the cause of the outcomes, it was not possible to rule out other potential confounders that could affect the prevalence of the diseases and the visits to health centres and hospitals.

It is important to keep in mind that ACSC are a proxy indicator of the performance of primary health services; while commonly accepted in the literature [13, 15], there might other, more direct measures of performance that were not possible to apply in this study. The diseases selected to obtain the ACSC were based on previous studies from the region and covered all age groups; however, attention should be paid when making comparison with studies that included other type of diseases and/or age-specific conditions. While case ascertainment and reporting can be an issue, it was not possible to assess the quality of the register used.

## Conclusion

The study revealed an important change in the pattern of ACSC hospitalization rates, from an increasing trend since the beginning of the 1990 s, peaking around the time of the intervention, to a decreasing trend following the 2008 constitutional health reform. A very similar pattern both in magnitude and direction of the ACSC rates was observed in both sexes. Given that the implemented health care reform seems to improve the performance of primary care services, a continuous strengthening of the primary care model in the country is recommended. Additionally, a regular monitoring of ACSC hospitalization rates in the coming years is advisable to ascertain whether the effects of the intervention are long lasting and sustainable. Finally, a health economic evaluation considering avoided hospitalizations and associated costs is suggested to allow a broader discussion of the sustainability of this primary health care model.

## Supplementary Information

**Additional file 1.**

**Additional file 2.**

**Additional file 3.**

## Data Availability

The data that support the findings of this study are publicly available at: https://www.ecuadorencifras.gob.ec/camas-y-egresos-hospitalarios/.
