# Supplementary material for: Assessing the impact of the 2008 health reform in Ecuador on the performance of primary health care services: an interrupted time series analysis
Source: Int J Equity Health. 2021 Jul 22;20:169. doi: 10.1186/s12939-021-01495-2 (PMC8296739; doi:10.1186/s12939-021-01495-2)
Supplement: Supplementary file 1 — Additional file 1. [file 12939_2021_1495_MOESM1_ESM.docx]

Figure 1S: Total hospitalization rates by sex, 1997 - 2018, Ecuador
