# Supplementary material for: Assessing the impact of the 2008 health reform in Ecuador on the performance of primary health care services: an interrupted time series analysis
Source: Int J Equity Health. 2021 Jul 22;20:169. doi: 10.1186/s12939-021-01495-2 (PMC8296739; doi:10.1186/s12939-021-01495-2)
Supplement: Supplementary file 2 — Additional file 2. [file 12939_2021_1495_MOESM2_ESM.pdf]

Table 1S. Raw data of ACSC by sex, year and agegroup

sex	year	agebracket	acsc	nonacsc	sumcases	pop	whopop
1	1997	0 to 4 years	7654	23673	31327	758038	88569
1	1997	5 to 9 years	3915	11848	15763	712768	86870
1	1997	10 to 14 years	1748	9258	11006	681306	85970
1	1997	15 to 19 years	928	11448	12376	624017	84670
1	1997	20 to 24 years	854	12531	13385	566382	82171
1	1997	25 to 29 years	825	11412	12237	506784	79272
1	1997	30 to 34 years	848	11146	11994	443925	76073
1	1997	35 to 39 years	802	10030	10832	378316	71475
1	1997	40 to 44 years	941	9092	10033	311122	65877
1	1997	45 to 49 years	969	7691	8660	260592	60379
1	1997	50 to 54 years	1042	7109	8151	203145	53681
1	1997	55 to 59 years	1099	6562	7661	161511	45484
1	1997	60 to 64 years	1307	6853	8160	130486	37187
1	1997	>65 years	5824	24448	30272	274306	82322
2	1997	0 to 4 years	6148	17744	23892	729941	88569
2	1997	5 to 9 years	2983	7982	10965	688411	86870
2	1997	10 to 14 years	1440	7629	9069	659736	85970
2	1997	15 to 19 years	1152	50196	51348	607101	84670
2	1997	20 to 24 years	1358	75260	76618	555899	82171
2	1997	25 to 29 years	1271	65983	67254	503422	79272
2	1997	30 to 34 years	1217	49461	50678	446891	76073
2	1997	35 to 39 years	1182	31742	32924	383376	71475
2	1997	40 to 44 years	1261	18228	19489	315185	65877
2	1997	45 to 49 years	1378	11651	13029	265033	60379
2	1997	50 to 54 years	1514	8742	10256	207432	53681
2	1997	55 to 59 years	1468	6720	8188	166666	45484
2	1997	60 to 64 years	1745	6811	8556	136923	37187
2	1997	>65 years	6615	20068	26683	313268	82322
1	1998	0 to 4 years	9076	22786	31862	758038	88569
1	1998	5 to 9 years	4458	12828	17286	712768	86870
1	1998	10 to 14 years	2143	9969	12112	681306	85970
1	1998	15 to 19 years	1025	12497	13522	624017	84670
1	1998	20 to 24 years	973	13245	14218	566382	82171
1	1998	25 to 29 years	1013	12328	13341	506784	79272
1	1998	30 to 34 years	938	11608	12546	443925	76073
1	1998	35 to 39 years	934	10906	11840	378316	71475
1	1998	40 to 44 years	1033	9723	10756	311122	65877
1	1998	45 to 49 years	1104	8452	9556	260592	60379
1	1998	50 to 54 years	1216	7708	8924	203145	53681
1	1998	55 to 59 years	1228	6985	8213	161511	45484
1	1998	60 to 64 years	1435	7544	8979	130486	37187
1	1998	>65 years	6567	26309	32876	274306	82322
2	1998	0 to 4 years	7398	18278	25676	729941	88569
2	1998	5 to 9 years	3536	8397	11933	688411	86870
2	1998	10 to 14 years	1683	8216	9899	659736	85970
2	1998	15 to 19 years	1202	51928	53130	607101	84670
2	1998	20 to 24 years	1503	78156	79659	555899	82171
2	1998	25 to 29 years	1388	66597	67985	503422	79272
2	1998	30 to 34 years	1360	49703	51063	446891	76073

Table 1S. Raw data of ACSC by sex, year and agegroup

2	1998	35 to 39 years	1301	32688	33989	383376	71475
2	1998	40 to 44 years	1355	18814	20169	315185	65877
2	1998	45 to 49 years	1519	12528	14047	265033	60379
2	1998	50 to 54 years	1629	9651	11280	207432	53681
2	1998	55 to 59 years	1589	7162	8751	166666	45484
2	1998	60 to 64 years	1868	7166	9034	136923	37187
2	1998	>65 years	7255	21656	28911	313268	82322
1	1999	0 to 4 years	7422	23967	31389	763597	88569
1	1999	5 to 9 years	3595	12589	16184	721616	86870
1	1999	10 to 14 years	1516	9872	11388	692267	85970
1	1999	15 to 19 years	590	10273	10863	634025	84670
1	1999	20 to 24 years	720	12271	12991	576888	82171
1	1999	25 to 29 years	681	11479	12160	518793	79272
1	1999	30 to 34 years	706	10874	11580	455949	76073
1	1999	35 to 39 years	718	10268	10986	389006	71475
1	1999	40 to 44 years	858	8957	9815	324129	65877
1	1999	45 to 49 years	999	7950	8949	271903	60379
1	1999	50 to 54 years	1066	7458	8524	209959	53681
1	1999	55 to 59 years	1096	6633	7729	166229	45484
1	1999	60 to 64 years	1294	7091	8385	134563	37187
1	1999	>65 years	6427	24943	31370	285280	82322
2	1999	0 to 4 years	6007	19428	25435	734982	88569
2	1999	5 to 9 years	2812	8570	11382	696612	86870
2	1999	10 to 14 years	1350	8446	9796	669997	85970
2	1999	15 to 19 years	1014	55608	56622	616630	84670
2	1999	20 to 24 years	1224	84469	85693	566230	82171
2	1999	25 to 29 years	1086	71846	72932	515665	79272
2	1999	30 to 34 years	1088	53154	54242	459507	76073
2	1999	35 to 39 years	1088	35193	36281	394645	71475
2	1999	40 to 44 years	1190	19481	20671	328740	65877
2	1999	45 to 49 years	1343	12537	13880	276833	60379
2	1999	50 to 54 years	1543	9183	10726	214503	53681
2	1999	55 to 59 years	1552	6949	8501	171555	45484
2	1999	60 to 64 years	1798	6728	8526	141233	37187
2	1999	>65 years	7388	21378	28766	325854	82322
1	2000	0 to 4 years	8738	24122	32860	769176	88569
1	2000	5 to 9 years	4596	13649	18245	730653	86870
1	2000	10 to 14 years	2019	12064	14083	703471	85970
1	2000	15 to 19 years	833	11368	12201	644276	84670
1	2000	20 to 24 years	825	13133	13958	587633	82171
1	2000	25 to 29 years	749	12047	12796	531125	79272
1	2000	30 to 34 years	795	11038	11833	468329	76073
1	2000	35 to 39 years	838	10492	11330	400035	71475
1	2000	40 to 44 years	966	9269	10235	337748	65877
1	2000	45 to 49 years	976	8207	9183	283768	60379
1	2000	50 to 54 years	1220	7886	9106	217044	53681
1	2000	55 to 59 years	1168	6848	8016	171111	45484
1	2000	60 to 64 years	1372	7379	8751	138779	37187
1	2000	>65 years	6625	26103	32728	296850	82322
2	2000	0 to 4 years	7280	18569	25849	740141	88569

Table 1S. Raw data of ACSC by sex, year and agegroup

2	2000	5 to 9 years	3782	9988	13770	704975	86870
2	2000	10 to 14 years	1770	10576	12346	680487	85970
2	2000	15 to 19 years	1129	57319	58448	626383	84670
2	2000	20 to 24 years	1381	86410	87791	576799	82171
2	2000	25 to 29 years	1266	73570	74836	528233	79272
2	2000	30 to 34 years	1256	55319	56575	472498	76073
2	2000	35 to 39 years	1208	36611	37819	406277	71475
2	2000	40 to 44 years	1348	20956	22304	342928	65877
2	2000	45 to 49 years	1478	13834	15312	289214	60379
2	2000	50 to 54 years	1834	10386	12220	221863	53681
2	2000	55 to 59 years	1713	7663	9376	176605	45484
2	2000	60 to 64 years	1880	7240	9120	145705	37187
2	2000	>65 years	7984	22834	30818	339104	82322
1	2001	0 to 4 years	8920	26469	35389	774881	88569
1	2001	5 to 9 years	4461	14295	18756	739886	86870
1	2001	10 to 14 years	1967	10724	12691	714935	85970
1	2001	15 to 19 years	999	11659	12658	654739	84670
1	2001	20 to 24 years	870	13238	14108	598647	82171
1	2001	25 to 29 years	825	11831	12656	543795	79272
1	2001	30 to 34 years	849	11209	12058	481097	76073
1	2001	35 to 39 years	843	10571	11414	411413	71475
1	2001	40 to 44 years	954	9629	10583	351976	65877
1	2001	45 to 49 years	1077	8566	9643	296214	60379
1	2001	50 to 54 years	1253	8159	9412	224421	53681
1	2001	55 to 59 years	1231	7028	8259	176156	45484
1	2001	60 to 64 years	1465	7519	8984	143140	37187
1	2001	>65 years	7029	26794	33823	309087	82322
2	2001	0 to 4 years	7275	20150	27425	745392	88569
2	2001	5 to 9 years	3642	10036	13678	713516	86870
2	2001	10 to 14 years	1777	9499	11276	691218	85970
2	2001	15 to 19 years	1248	58367	59615	636339	84670
2	2001	20 to 24 years	1431	88401	89832	587605	82171
2	2001	25 to 29 years	1307	72087	73394	541137	79272
2	2001	30 to 34 years	1272	54987	56259	485891	76073
2	2001	35 to 39 years	1240	36910	38150	418284	71475
2	2001	40 to 44 years	1328	21108	22436	357776	65877
2	2001	45 to 49 years	1497	14126	15623	302205	60379
2	2001	50 to 54 years	1933	10714	12647	229512	53681
2	2001	55 to 59 years	1805	7789	9594	181830	45484
2	2001	60 to 64 years	1991	7242	9233	150341	37187
2	2001	>65 years	8543	23572	32115	352890	82322
1	2002	0 to 4 years	9958	26818	36776	798492	88569
1	2002	5 to 9 years	4692	14596	19288	768450	86870
1	2002	10 to 14 years	2242	11895	14137	740203	85970
1	2002	15 to 19 years	952	11227	12179	678562	84670
1	2002	20 to 24 years	851	13225	14076	619981	82171
1	2002	25 to 29 years	837	11612	12449	563121	79272
1	2002	30 to 34 years	849	11058	11907	498966	76073
1	2002	35 to 39 years	849	10335	11184	429419	71475
1	2002	40 to 44 years	978	9649	10627	370424	65877

Table 1S. Raw data of ACSC by sex, year and agegroup

1	2002	45 to 49 years	1073	8612	9685	314712	60379
1	2002	50 to 54 years	1337	8692	10029	243526	53681
1	2002	55 to 59 years	1261	7270	8531	192460	45484
1	2002	60 to 64 years	1411	7669	9080	155451	37187
1	2002	>65 years	7174	28325	35499	338742	82322
2	2002	0 to 4 years	8477	20351	28828	741713	88569
2	2002	5 to 9 years	3932	10094	14026	706953	86870
2	2002	10 to 14 years	1902	10449	12351	682870	85970
2	2002	15 to 19 years	1159	63566	64725	629650	84670
2	2002	20 to 24 years	1346	95108	96454	581758	82171
2	2002	25 to 29 years	1252	74154	75406	536200	79272
2	2002	30 to 34 years	1241	56345	57586	482382	76073
2	2002	35 to 39 years	1165	37013	38178	417171	71475
2	2002	40 to 44 years	1431	21764	23195	358423	65877
2	2002	45 to 49 years	1686	13870	15556	304204	60379
2	2002	50 to 54 years	1978	10697	12675	234432	53681
2	2002	55 to 59 years	1942	8174	10116	186789	45484
2	2002	60 to 64 years	2116	7670	9786	154165	37187
2	2002	>65 years	8869	24627	33496	364308	82322
1	2003	0 to 4 years	11211	29418	40629	807808	88569
1	2003	5 to 9 years	4851	14989	19840	778442	86870
1	2003	10 to 14 years	2174	10033	12207	747938	85970
1	2003	15 to 19 years	779	10356	11135	685957	84670
1	2003	20 to 24 years	699	10395	11094	626003	82171
1	2003	25 to 29 years	745	12650	13395	568312	79272
1	2003	30 to 34 years	636	9164	9800	504130	76073
1	2003	35 to 39 years	768	10770	11538	435371	71475
1	2003	40 to 44 years	683	8711	9394	376970	65877
1	2003	45 to 49 years	1042	9668	10710	321883	60379
1	2003	50 to 54 years	919	7028	7947	252216	53681
1	2003	55 to 59 years	1350	8904	10254	200290	45484
1	2003	60 to 64 years	1359	8068	9427	161497	37187
1	2003	>65 years	9298	37844	47142	354336	82322
2	2003	0 to 4 years	9484	22344	31828	750016	88569
2	2003	5 to 9 years	4007	10917	14924	715803	86870
2	2003	10 to 14 years	1799	7427	9226	690011	85970
2	2003	15 to 19 years	1081	49132	50213	637165	84670
2	2003	20 to 24 years	1041	74908	75949	588890	82171
2	2003	25 to 29 years	1311	82992	84303	543584	79272
2	2003	30 to 34 years	988	51671	52659	490324	76073
2	2003	35 to 39 years	1129	46789	47918	425657	71475
2	2003	40 to 44 years	1052	27064	28116	366945	65877
2	2003	45 to 49 years	1375	19374	20749	312596	60379
2	2003	50 to 54 years	1282	11364	12646	243663	53681
2	2003	55 to 59 years	2123	11524	13647	194749	45484
2	2003	60 to 64 years	2043	8825	10868	160162	37187
2	2003	>65 years	11621	34315	45936	378857	82322
1	2004	0 to 4 years	12294	30831	43125	817269	88569
1	2004	5 to 9 years	5175	15436	20611	788569	86870
1	2004	10 to 14 years	2289	12742	15031	755773	85970

Table 1S. Raw data of ACSC by sex, year and agegroup

1	2004	15 to 19 years	830	11860	12690	693444	84670
1	2004	20 to 24 years	869	13774	14643	632126	82171
1	2004	25 to 29 years	865	12706	13571	573544	79272
1	2004	30 to 34 years	790	11902	12692	509346	76073
1	2004	35 to 39 years	835	10908	11743	441427	71475
1	2004	40 to 44 years	1088	10646	11734	383643	65877
1	2004	45 to 49 years	1201	9587	10788	329203	60379
1	2004	50 to 54 years	1572	9640	11212	261215	53681
1	2004	55 to 59 years	1430	8606	10036	208463	45484
1	2004	60 to 64 years	1649	8648	10297	167800	37187
1	2004	>65 years	8378	32282	40660	370909	82322
2	2004	0 to 4 years	10451	25157	35608	758478	88569
2	2004	5 to 9 years	4269	11458	15727	724817	86870
2	2004	10 to 14 years	2153	11947	14100	697262	85970
2	2004	15 to 19 years	1244	66096	67340	644822	84670
2	2004	20 to 24 years	1367	96862	98229	596146	82171
2	2004	25 to 29 years	1235	76217	77452	551086	79272
2	2004	30 to 34 years	1202	56368	57570	498425	76073
2	2004	35 to 39 years	1244	37385	38629	434328	71475
2	2004	40 to 44 years	1436	23320	24756	375683	65877
2	2004	45 to 49 years	1675	15850	17525	321238	60379
2	2004	50 to 54 years	2323	12670	14993	253284	53681
2	2004	55 to 59 years	2213	9689	11902	203060	45484
2	2004	60 to 64 years	2291	8543	10834	166397	37187
2	2004	>65 years	10938	29207	40145	394118	82322
1	2005	0 to 4 years	12587	33864	46451	813152	88569
1	2005	5 to 9 years	5542	16405	21947	780062	86870
1	2005	10 to 14 years	2473	13122	15595	744673	85970
1	2005	15 to 19 years	1031	12582	13613	682611	84670
1	2005	20 to 24 years	1089	14718	15807	621326	82171
1	2005	25 to 29 years	972	13927	14899	562682	79272
1	2005	30 to 34 years	969	12034	13003	499024	76073
1	2005	35 to 39 years	962	11356	12318	432828	71475
1	2005	40 to 44 years	1062	11059	12121	376518	65877
1	2005	45 to 49 years	1382	9793	11175	323489	60379
1	2005	50 to 54 years	1576	9646	11222	258311	53681
1	2005	55 to 59 years	1652	8928	10580	206869	45484
1	2005	60 to 64 years	1679	8947	10626	166812	37187
1	2005	>65 years	9422	34669	44091	373662	82322
2	2005	0 to 4 years	10664	26099	36763	779706	88569
2	2005	5 to 9 years	4611	11778	16389	749850	86870
2	2005	10 to 14 years	2345	12199	14544	720213	85970
2	2005	15 to 19 years	1372	69823	71195	667073	84670
2	2005	20 to 24 years	1539	99146	100685	616871	82171
2	2005	25 to 29 years	1456	80987	82443	571275	79272
2	2005	30 to 34 years	1416	57751	59167	518169	76073
2	2005	35 to 39 years	1398	38946	40344	453080	71475
2	2005	40 to 44 years	1611	24113	25724	393075	65877
2	2005	45 to 49 years	1932	16194	18126	337100	60379
2	2005	50 to 54 years	2451	13119	15570	268161	53681

Table 1S. Raw data of ACSC by sex, year and agegroup

2	2005	55 to 59 years	2324	10528	12852	215126	45484
2	2005	60 to 64 years	2458	9456	11914	175268	37187
2	2005	>65 years	12264	31515	43779	414311	82322
1	2006	0 to 4 years	14184	36127	50311	823220	88569
1	2006	5 to 9 years	6490	18507	24997	790613	86870
1	2006	10 to 14 years	3022	13659	16681	752452	85970
1	2006	15 to 19 years	1070	13115	14185	689817	84670
1	2006	20 to 24 years	1081	15769	16850	627224	82171
1	2006	25 to 29 years	1042	15055	16097	567606	79272
1	2006	30 to 34 years	980	12957	13937	503668	76073
1	2006	35 to 39 years	994	12049	13043	438382	71475
1	2006	40 to 44 years	1196	11814	13010	382944	65877
1	2006	45 to 49 years	1302	10661	11963	330728	60379
1	2006	50 to 54 years	1651	10339	11990	267627	53681
1	2006	55 to 59 years	1751	9844	11595	215424	45484
1	2006	60 to 64 years	1829	9581	11410	173367	37187
1	2006	>65 years	10339	35910	46249	392200	82322
2	2006	0 to 4 years	12027	28016	40043	788710	88569
2	2006	5 to 9 years	5534	13433	18967	759380	86870
2	2006	10 to 14 years	2638	12720	15358	727787	85970
2	2006	15 to 19 years	1597	74377	75974	675065	84670
2	2006	20 to 24 years	1848	104905	106753	624515	82171
2	2006	25 to 29 years	1651	87884	89535	579148	79272
2	2006	30 to 34 years	1582	62195	63777	526635	76073
2	2006	35 to 39 years	1461	41985	43446	462259	71475
2	2006	40 to 44 years	1714	25526	27240	402475	65877
2	2006	45 to 49 years	2002	17604	19606	346486	60379
2	2006	50 to 54 years	2590	14120	16710	278890	53681
2	2006	55 to 59 years	2604	11371	13975	224419	45484
2	2006	60 to 64 years	2652	9933	12585	182164	37187
2	2006	>65 years	13069	33681	46750	431401	82322
1	2007	0 to 4 years	17083	33814	50897	833498	88569
1	2007	5 to 9 years	8115	21126	29241	801368	86870
1	2007	10 to 14 years	3374	15775	19149	760359	85970
1	2007	15 to 19 years	1122	14870	15992	697127	84670
1	2007	20 to 24 years	1108	16952	18060	633222	82171
1	2007	25 to 29 years	1073	16768	17841	572616	79272
1	2007	30 to 34 years	1050	14210	15260	508376	76073
1	2007	35 to 39 years	1025	12527	13552	444019	71475
1	2007	40 to 44 years	1252	12635	13887	389483	65877
1	2007	45 to 49 years	1369	11327	12696	338134	60379
1	2007	50 to 54 years	1803	11379	13182	277305	53681
1	2007	55 to 59 years	1850	10679	12529	224345	45484
1	2007	60 to 64 years	2003	10319	12322	180188	37187
1	2007	>65 years	10700	39792	50492	411844	82322
2	2007	0 to 4 years	14374	25704	40078	797891	88569
2	2007	5 to 9 years	6640	15173	21813	769095	86870
2	2007	10 to 14 years	2970	14504	17474	735487	85970
2	2007	15 to 19 years	1760	78909	80669	683185	84670
2	2007	20 to 24 years	1867	109167	111034	632269	82171

Table 1S. Raw data of ACSC by sex, year and agegroup

2	2007	25 to 29 years	1789	92558	94347	587173	79272
2	2007	30 to 34 years	1551	64601	66152	535256	76073
2	2007	35 to 39 years	1561	43816	45377	471656	71475
2	2007	40 to 44 years	1888	27079	28967	412109	65877
2	2007	45 to 49 years	2126	19212	21338	356139	60379
2	2007	50 to 54 years	2654	15579	18233	290080	53681
2	2007	55 to 59 years	2774	12340	15114	234142	45484
2	2007	60 to 64 years	2865	11099	13964	189344	37187
2	2007	>65 years	13782	36605	50387	449272	82322
1	2008	0 to 4 years	16682	41049	57731	843981	88569
1	2008	5 to 9 years	7507	21950	29457	812346	86870
1	2008	10 to 14 years	3192	15690	18882	768418	85970
1	2008	15 to 19 years	1139	15847	16986	704540	84670
1	2008	20 to 24 years	1157	18140	19297	639311	82171
1	2008	25 to 29 years	1161	18246	19407	577721	79272
1	2008	30 to 34 years	1113	15590	16703	513157	76073
1	2008	35 to 39 years	1066	13718	14784	449749	71475
1	2008	40 to 44 years	1349	13418	14767	396159	65877
1	2008	45 to 49 years	1581	12191	13772	345719	60379
1	2008	50 to 54 years	2049	12313	14362	287379	53681
1	2008	55 to 59 years	2101	11983	14084	233666	45484
1	2008	60 to 64 years	2238	11389	13627	187310	37187
1	2008	>65 years	11843	42464	54307	432672	82322
2	2008	0 to 4 years	13783	31285	45068	807248	88569
2	2008	5 to 9 years	6313	15710	22023	778998	86870
2	2008	10 to 14 years	2941	15108	18049	743319	85970
2	2008	15 to 19 years	1742	83382	85124	691444	84670
2	2008	20 to 24 years	2014	113002	115016	640172	82171
2	2008	25 to 29 years	1960	97043	99003	595327	79272
2	2008	30 to 34 years	1602	69372	70974	544046	76073
2	2008	35 to 39 years	1628	46199	47827	481256	71475
2	2008	40 to 44 years	1914	29035	30949	421994	65877
2	2008	45 to 49 years	2413	21070	23483	366092	60379
2	2008	50 to 54 years	3064	17111	20175	301751	53681
2	2008	55 to 59 years	3070	13439	16509	244309	45484
2	2008	60 to 64 years	3178	12060	15238	196819	37187
2	2008	>65 years	15509	40173	55682	467978	82322
1	2009	0 to 4 years	15774	43749	59523	854686	88569
1	2009	5 to 9 years	7265	23094	30359	823536	86870
1	2009	10 to 14 years	3236	17257	20493	776608	85970
1	2009	15 to 19 years	1194	16280	17474	712046	84670
1	2009	20 to 24 years	1332	19005	20337	645504	82171
1	2009	25 to 29 years	1406	19260	20666	582907	79272
1	2009	30 to 34 years	1395	16845	18240	518011	76073
1	2009	35 to 39 years	1364	14919	16283	455564	71475
1	2009	40 to 44 years	1541	14275	15816	402953	65877
1	2009	45 to 49 years	1836	13232	15068	353486	60379
1	2009	50 to 54 years	2208	12794	15002	297833	53681
1	2009	55 to 59 years	2290	12402	14692	243391	45484
1	2009	60 to 64 years	2467	12310	14777	194724	37187

Table 1S. Raw data of ACSC by sex, year and agegroup

1	2009	>65 years	12335	45090	57425	454771	82322
2	2009	0 to 4 years	13586	33223	46809	816789	88569
2	2009	5 to 9 years	6478	16561	23039	789091	86870
2	2009	10 to 14 years	3036	16711	19747	751301	85970
2	2009	15 to 19 years	1949	86687	88636	699825	84670
2	2009	20 to 24 years	2158	116954	119112	648214	82171
2	2009	25 to 29 years	2106	99797	101903	603645	79272
2	2009	30 to 34 years	1902	73048	74950	553015	76073
2	2009	35 to 39 years	1864	48444	50308	491074	71475
2	2009	40 to 44 years	2065	30331	32396	432137	65877
2	2009	45 to 49 years	2634	22195	24829	376337	60379
2	2009	50 to 54 years	3243	17867	21110	313931	53681
2	2009	55 to 59 years	3340	14443	17783	254951	45484
2	2009	60 to 64 years	3332	13000	16332	204608	37187
2	2009	>65 years	15955	42893	58848	487534	82322
1	2010	0 to 4 years	17760	46735	64495	865609	88569
1	2010	5 to 9 years	7986	23962	31948	834951	86870
1	2010	10 to 14 years	3556	18606	22162	784952	85970
1	2010	15 to 19 years	1237	17406	18643	719672	84670
1	2010	20 to 24 years	1262	20147	21409	651794	82171
1	2010	25 to 29 years	1328	20774	22102	588187	79272
1	2010	30 to 34 years	1354	18541	19895	522934	76073
1	2010	35 to 39 years	1227	16180	17407	461463	71475
1	2010	40 to 44 years	1499	15198	16697	409876	65877
1	2010	45 to 49 years	1765	14548	16313	361444	60379
1	2010	50 to 54 years	2160	14086	16246	308705	53681
1	2010	55 to 59 years	2409	13986	16395	253563	45484
1	2010	60 to 64 years	2606	13574	16180	202462	37187
1	2010	>65 years	13476	50156	63632	478263	82322
2	2010	0 to 4 years	15327	37564	52891	826528	88569
2	2010	5 to 9 years	7018	17485	24503	799393	86870
2	2010	10 to 14 years	3317	17628	20945	759419	85970
2	2010	15 to 19 years	1781	87195	88976	708354	84670
2	2010	20 to 24 years	2050	116359	118409	656390	82171
2	2010	25 to 29 years	2080	101975	104055	612107	79272
2	2010	30 to 34 years	1957	77373	79330	562150	76073
2	2010	35 to 39 years	1848	50549	52397	501112	71475
2	2010	40 to 44 years	2091	31687	33778	442537	65877
2	2010	45 to 49 years	2596	23868	26464	386889	60379
2	2010	50 to 54 years	3165	18669	21834	326634	53681
2	2010	55 to 59 years	3427	15774	19201	266079	45484
2	2010	60 to 64 years	3535	14467	18002	212730	37187
2	2010	>65 years	17451	48503	65954	508031	82322
1	2011	0 to 4 years	16265	49020	65285	866849	88569
1	2011	5 to 9 years	7165	24473	31638	842279	86870
1	2011	10 to 14 years	3319	19153	22472	795867	85970
1	2011	15 to 19 years	1225	18559	19784	732101	84670
1	2011	20 to 24 years	1247	21711	22958	662920	82171
1	2011	25 to 29 years	1275	21969	23244	599205	79272
1	2011	30 to 34 years	1342	19929	21271	535044	76073

Table 1S. Raw data of ACSC by sex, year and agegroup

1	2011	35 to 39 years	1340	16867	18207	472396	71475
1	2011	40 to 44 years	1567	15913	17480	418872	65877
1	2011	45 to 49 years	1850	15179	17029	370223	60379
1	2011	50 to 54 years	2270	14824	17094	317984	53681
1	2011	55 to 59 years	2595	14621	17216	262088	45484
1	2011	60 to 64 years	2848	14564	17412	209118	37187
1	2011	>65 years	14708	52936	67644	482730	82322
2	2011	0 to 4 years	14220	37852	52072	827967	88569
2	2011	5 to 9 years	6472	17814	24286	805607	86870
2	2011	10 to 14 years	2900	17870	20770	767937	85970
2	2011	15 to 19 years	1824	91738	93562	718139	84670
2	2011	20 to 24 years	2063	119772	121835	664821	82171
2	2011	25 to 29 years	2095	106582	108677	620243	79272
2	2011	30 to 34 years	1984	82213	84197	573207	76073
2	2011	35 to 39 years	1733	52698	54431	513403	71475
2	2011	40 to 44 years	2078	32941	35019	453442	65877
2	2011	45 to 49 years	2568	24840	27408	397764	60379
2	2011	50 to 54 years	3344	19866	23210	338174	53681
2	2011	55 to 59 years	3604	17128	20732	276578	45484
2	2011	60 to 64 years	3685	15335	19020	221047	37187
2	2011	>65 years	18768	50835	69603	520426	82322
1	2012	0 to 4 years	15695	49006	64701	866450	88569
1	2012	5 to 9 years	7664	25399	33063	848885	86870
1	2012	10 to 14 years	3438	20803	24241	806206	85970
1	2012	15 to 19 years	1256	19385	20641	744368	84670
1	2012	20 to 24 years	1234	21947	23181	674309	82171
1	2012	25 to 29 years	1347	21903	23250	610081	79272
1	2012	30 to 34 years	1303	20809	22112	547126	76073
1	2012	35 to 39 years	1350	17941	19291	483711	71475
1	2012	40 to 44 years	1530	16454	17984	428131	65877
1	2012	45 to 49 years	1850	15662	17512	378933	60379
1	2012	50 to 54 years	2367	15452	17819	327186	53681
1	2012	55 to 59 years	2709	15167	17876	270832	45484
1	2012	60 to 64 years	2819	15197	18016	216103	37187
1	2012	>65 years	15190	54960	70150	489591	82322
2	2012	0 to 4 years	13835	37761	51596	828008	88569
2	2012	5 to 9 years	6949	18575	25524	811352	86870
2	2012	10 to 14 years	3333	19597	22930	776061	85970
2	2012	15 to 19 years	1937	93005	94942	727743	84670
2	2012	20 to 24 years	2202	117449	119651	673635	82171
2	2012	25 to 29 years	2082	104417	106499	628162	79272
2	2012	30 to 34 years	2049	83771	85820	583546	76073
2	2012	35 to 39 years	1922	54688	56610	525760	71475
2	2012	40 to 44 years	2090	34051	36141	464654	65877
2	2012	45 to 49 years	2610	25260	27870	408484	60379
2	2012	50 to 54 years	3354	20953	24307	349623	53681
2	2012	55 to 59 years	3584	17866	21450	287405	45484
2	2012	60 to 64 years	3779	15949	19728	229794	37187
2	2012	>65 years	19583	53749	73332	534834	82322
1	2013	0 to 4 years	16324	48773	65097	864669	88569

Table 1S. Raw data of ACSC by sex, year and agegroup

1	2013	5 to 9 years	7534	25955	33489	854691	86870
1	2013	10 to 14 years	3437	20859	24296	815838	85970
1	2013	15 to 19 years	1244	20010	21254	756376	84670
1	2013	20 to 24 years	1350	23371	24721	685997	82171
1	2013	25 to 29 years	1429	23853	25282	620881	79272
1	2013	30 to 34 years	1517	22513	24030	559055	76073
1	2013	35 to 39 years	1487	19505	20992	495340	71475
1	2013	40 to 44 years	1628	17921	19549	437744	65877
1	2013	45 to 49 years	2043	17018	19061	387618	60379
1	2013	50 to 54 years	2453	16842	19295	336267	53681
1	2013	55 to 59 years	2811	16433	19244	279746	45484
1	2013	60 to 64 years	2993	16714	19707	223411	37187
1	2013	>65 years	15432	59690	75122	498302	82322
2	2013	0 to 4 years	14798	38785	53583	826731	88569
2	2013	5 to 9 years	7075	18771	25846	816503	86870
2	2013	10 to 14 years	3520	19895	23415	783725	85970
2	2013	15 to 19 years	1944	88917	90861	737082	84670
2	2013	20 to 24 years	2170	111312	113482	682849	82171
2	2013	25 to 29 years	2188	101571	103759	635987	79272
2	2013	30 to 34 years	2100	83603	85703	593148	76073
2	2013	35 to 39 years	2007	55897	57904	538054	71475
2	2013	40 to 44 years	2097	35668	37765	476215	65877
2	2013	45 to 49 years	2595	26165	28760	419090	60379
2	2013	50 to 54 years	3300	22169	25469	360935	53681
2	2013	55 to 59 years	3617	19313	22930	298503	45484
2	2013	60 to 64 years	3763	16821	20584	238973	37187
2	2013	>65 years	19295	58494	77789	551019	82322
1	2014	0 to 4 years	15713	50665	66378	862157	88569
1	2014	5 to 9 years	7217	26072	33289	859265	86870
1	2014	10 to 14 years	3295	21552	24847	824704	85970
1	2014	15 to 19 years	1076	19874	20950	768031	84670
1	2014	20 to 24 years	1164	23251	24415	697957	82171
1	2014	25 to 29 years	1341	24531	25872	631695	79272
1	2014	30 to 34 years	1333	22710	24043	570725	76073
1	2014	35 to 39 years	1447	20651	22098	507231	71475
1	2014	40 to 44 years	1558	18345	19903	447786	65877
1	2014	45 to 49 years	1803	17437	19240	396328	60379
1	2014	50 to 54 years	2396	17504	19900	345188	53681
1	2014	55 to 59 years	2676	17162	19838	288760	45484
1	2014	60 to 64 years	2969	17476	20445	231040	37187
1	2014	>65 years	16005	60738	76743	508685	82322
2	2014	0 to 4 years	14387	43366	57753	824642	88569
2	2014	5 to 9 years	7089	18643	25732	820708	86870
2	2014	10 to 14 years	3338	20229	23567	790883	85970
2	2014	15 to 19 years	1779	89809	91588	746133	84670
2	2014	20 to 24 years	1969	109578	111547	692359	82171
2	2014	25 to 29 years	2173	101738	103911	643855	79272
2	2014	30 to 34 years	2009	83241	85250	602107	76073
2	2014	35 to 39 years	1891	57228	59119	550125	71475
2	2014	40 to 44 years	2065	35606	37671	488087	65877

Table 1S. Raw data of ACSC by sex, year and agegroup

2	2014	45 to 49 years	2614	26322	28936	429656	60379
2	2014	50 to 54 years	3295	22410	25705	372082	53681
2	2014	55 to 59 years	3496	19711	23207	309801	45484
2	2014	60 to 64 years	3867	18194	22061	248574	37187
2	2014	>65 years	19825	58916	78741	568902	82322
1	2015	0 to 4 years	14771	49339	64110	859360	88569
1	2015	5 to 9 years	7042	26326	33368	862323	86870
1	2015	10 to 14 years	3090	20317	23407	832792	85970
1	2015	15 to 19 years	1108	19928	21036	756376	84670
1	2015	20 to 24 years	1112	23277	24389	710096	82171
1	2015	25 to 29 years	1195	23659	24854	642583	79272
1	2015	30 to 34 years	1261	22327	23588	582098	76073
1	2015	35 to 39 years	1365	20433	21798	519334	71475
1	2015	40 to 44 years	1491	18135	19626	458294	65877
1	2015	45 to 49 years	1802	16997	18799	405129	60379
1	2015	50 to 54 years	2264	16940	19204	353969	53681
1	2015	55 to 59 years	2683	17245	19928	297828	45484
1	2015	60 to 64 years	2927	17620	20547	238963	37187
1	2015	>65 years	15765	61958	77723	520559	82322
2	2015	0 to 4 years	13838	39364	53202	822154	88569
2	2015	5 to 9 years	7167	19170	26337	823662	86870
2	2015	10 to 14 years	3186	18702	21888	797560	85970
2	2015	15 to 19 years	1617	84528	86145	754881	84670
2	2015	20 to 24 years	1853	105710	107563	701972	82171
2	2015	25 to 29 years	2006	98440	100446	651894	79272
2	2015	30 to 34 years	1892	81332	83224	610580	76073
2	2015	35 to 39 years	1928	56465	58393	561778	71475
2	2015	40 to 44 years	1986	33821	35807	500202	65877
2	2015	45 to 49 years	2426	24908	27334	440291	60379
2	2015	50 to 54 years	3190	21619	24809	383063	53681
2	2015	55 to 59 years	3427	19028	22455	321207	45484
2	2015	60 to 64 years	3690	18390	22080	258558	37187
2	2015	>65 years	19442	59542	78984	588432	82322
1	2016	0 to 4 years	16527	48733	65260	856552	88569
1	2016	5 to 9 years	7950	25613	33563	863753	86870
1	2016	10 to 14 years	3287	19331	22618	840132	85970
1	2016	15 to 19 years	1011	18723	19734	790072	84670
1	2016	20 to 24 years	1114	21878	22992	722223	82171
1	2016	25 to 29 years	1133	22120	23253	653642	79272
1	2016	30 to 34 years	1289	21165	22454	593207	76073
1	2016	35 to 39 years	1399	19371	20770	531540	71475
1	2016	40 to 44 years	1485	16849	18334	469240	65877
1	2016	45 to 49 years	1754	15858	17612	414094	60379
1	2016	50 to 54 years	2277	16496	18773	362641	53681
1	2016	55 to 59 years	2678	16538	19216	306871	45484
1	2016	60 to 64 years	3037	17440	20477	247138	37187
1	2016	>65 years	15747	61520	77267	533865	82322
2	2016	0 to 4 years	15395	38739	54134	819467	88569
2	2016	5 to 9 years	7979	18691	26670	825270	86870
2	2016	10 to 14 years	3353	17517	20870	803791	85970

Table 1S. Raw data of ACSC by sex, year and agegroup

2	2016	15 to 19 years	1707	76959	78666	763288	84670
2	2016	20 to 24 years	1772	100897	102669	711538	82171
2	2016	25 to 29 years	1854	95817	97671	660237	79272
2	2016	30 to 34 years	1946	80307	82253	618713	76073
2	2016	35 to 39 years	1974	55650	57624	572831	71475
2	2016	40 to 44 years	1933	32715	34648	512473	65877
2	2016	45 to 49 years	2415	23273	25688	451147	60379
2	2016	50 to 54 years	3036	20626	23662	393873	53681
2	2016	55 to 59 years	3393	18587	21980	332617	45484
2	2016	60 to 64 years	3509	17297	20806	268886	37187
2	2016	>65 years	19858	58482	78340	609629	82322
1	2017	0 to 4 years	18891	56672	75563	853975	88569
1	2017	5 to 9 years	4135	20042	24177	863498	86870
1	2017	10 to 14 years	1769	17508	19277	846765	85970
1	2017	15 to 19 years	1021	18850	19871	800288	84670
1	2017	20 to 24 years	1054	21273	22327	734190	82171
1	2017	25 to 34 years	2330	42547	44877	1269104	155345
1	2017	35 to 44 years	2671	36615	39286	1024291	137352
1	2017	45 to 54 years	3934	32523	36457	794563	114060
1	2017	55 to 64 years	5617	34812	40429	571375	82671
1	2017	>65 years	16883	63829	80712	548508	82322
2	2017	0 to 4 years	17359	45940	63299	816875	88569
2	2017	5 to 9 years	4810	13861	18671	825425	86870
2	2017	10 to 14 years	1978	15719	17697	809561	85970
2	2017	15 to 19 years	1700	77693	79393	771299	84670
2	2017	20 to 24 years	1895	104210	106105	720924	82171
2	2017	25 to 34 years	3822	181046	184868	1295584	155345
2	2017	35 to 44 years	3764	90166	93930	1107968	137352
2	2017	45 to 54 years	5216	44601	49817	866853	114060
2	2017	55 to 64 years	6911	36850	43761	623495	82671
2	2017	>65 years	21258	61985	83243	632436	82322
1	2018	0 to 4 years	18230	54645	72875	851631	88569
1	2018	5 to 9 years	3937	19242	23179	861847	86870
1	2018	10 to 14 years	1648	16799	18447	852613	85970
1	2018	15 to 19 years	955	19295	20250	809815	84670
1	2018	20 to 24 years	969	21159	22128	745908	82171
1	2018	25 to 29 years	1114	22534	23648	676519	79272
1	2018	30 to 34 years	1214	21428	22642	615017	76073
1	2018	35 to 39 years	1367	20137	21504	555764	71475
1	2018	40 to 44 years	1438	18095	19533	492203	65877
1	2018	45 to 49 years	1734	16804	18538	432867	60379
1	2018	50 to 54 years	2219	17051	19270	379847	53681
1	2018	55 to 59 years	2809	18391	21200	324722	45484
1	2018	60 to 64 years	3140	18897	22037	264069	37187
1	2018	>65 years	17683	67158	84841	564439	82322
2	2018	0 to 4 years	17230	44196	61426	814517	88569
2	2018	5 to 9 years	4536	13269	17805	824252	86870
2	2018	10 to 14 years	1888	15107	16995	814748	85970
2	2018	15 to 19 years	1600	77368	78968	778853	84670
2	2018	20 to 24 years	1708	104086	105794	730047	82171

Table 1S. Raw data of ACSC by sex, year and agegroup

2	2018	25 to 29 years	1768	101722	103490	678067	79272
2	2018	30 to 34 years	1859	84430	86289	634428	76073
2	2018	35 to 39 years	1906	60025	61931	592800	71475
2	2018	40 to 44 years	1960	34739	36699	537058	65877
2	2018	45 to 49 years	2273	24806	27079	473856	60379
2	2018	50 to 54 years	2913	21634	24547	415052	53681
2	2018	55 to 59 years	3674	19439	23113	355158	45484
2	2018	60 to 64 years	3474	18991	22465	290464	37187
2	2018	>65 years	21283	66682	87965	656847	82322
