# Supplementary material for: Assessing the impact of the 2008 health reform in Ecuador on the performance of primary health care services: an interrupted time series analysis
Source: Int J Equity Health. 2021 Jul 22;20:169. doi: 10.1186/s12939-021-01495-2 (PMC8296739; doi:10.1186/s12939-021-01495-2)
Supplement: Supplementary file 3 — Additional file 3. [file 12939_2021_1495_MOESM3_ESM.docx]

Table 2S. Total absolute and relative number of ACSC hospital admissions per age groups, 1997-2016, 2018

|  | 1997 | 1998 | 1999 | 2000 | 2001 | 2002 | 2003 | 2004 | 2005 | 2006 | 2007 | 2008 |
| --- | --- | --- | --- | --- | --- | --- | --- | --- | --- | --- | --- | --- |
| 0 to 4 years | 13802 (25%) | 16474 (28.6%) | 13429 (23.6%) | 16018 (27.3%) | 16195 (25.8%) | 18435 (28.1%) | 20695 (28.6%) | 22745 (28.9%) | 23251 (27.9%) | 26211 (29%) | 31457 (34.6%) | 30465 (29.6%) |
| 5 to 9 years | 6898 (25.8%) | 7994 (27.4%) | 6407 (23.2%) | 8378 (26.2%) | 8103 (25%) | 8624 (25.9%) | 8858 (25.5%) | 9444 (26%) | 10153 (26.5%) | 12024 (27.3%) | 14755 (28.9%) | 13820 (26.8%) |
| 10 to 14 years | 3188 (15.9%) | 3826 (17.4%) | 2866 (13.5%) | 3789 (14.3%) | 3744 (15.6%) | 4144 (15.6%) | 3973 (18.5%) | 4442 (15.2%) | 4818 (16%) | 5660 (17.7%) | 6344 (17.3%) | 6133 (16.6%) |
| 15 to 19 years | 2080 (3.3%) | 2227 (3.3%) | 1604 (2.4%) | 1962 (2.8%) | 2247 (3.1%) | 2111 (2.7%) | 1860 (3%) | 2074 (2.6%) | 2403 (2.8%) | 2667 (3%) | 2882 (3%) | 2881 (2.8%) |
| 20 to 24 years | 2212 (2.5%) | 2476 (2.6%) | 1944 (2%) | 2206 (2.2%) | 2301 (2.2%) | 2197 (2%) | 1740 (2%) | 2236 (2%) | 2628 (2.3%) | 2929 (2.4%) | 2975 (2.3%) | 3171 (2.4%) |
| 25 to 29 years | 2096 (2.6%) | 2401 (3%) | 1767 (2.1%) | 2015 (2.3%) | 2132 (2.5%) | 2089 (2.4%) | 2056 (2.1%) | 2100 (2.3%) | 2428 (2.5%) | 2693 (2.5%) | 2862 (2.6%) | 3121 (2.6%) |
| 30 to 34 years | 2065 (3.3%) | 2298 (3.6%) | 1794 (2.7%) | 2051 (3%) | 2121 (3.1%) | 2090 (3%) | 1624 (2.6%) | 1992 (2.8%) | 2385 (3.3%) | 2562 (3.3%) | 2601 (3.2%) | 2715 (3.1%) |
| 35 to 39 years | 1984 (4.5%) | 2235 (4.9%) | 1806 (3.8%) | 2046 (4.2%) | 2083 (4.2%) | 2014 (4.1%) | 1897 (3.2%) | 2079 (4.1%) | 2360 (4.5%) | 2455 (4.3%) | 2586 (4.4%) | 2694 (4.3%) |
| 40 to 44 years | 2202 (7.5%) | 2388 (7.7%) | 2048 (6.7%) | 2314 (7.1%) | 2282 (6.9%) | 2409 (7.1%) | 1735 (4.6%) | 2524 (6.9%) | 2673 (7.1%) | 2910 (7.2%) | 3140 (7.3%) | 3263 (7.1%) |
| 45 to 49 years | 2347 (10.8%) | 2623 (11.1%) | 2342 (10.3%) | 2454 (10%) | 2574 (10.2%) | 2759 (10.9%) | 2417 (7.7%) | 2876 (10.2%) | 3314 (11.3%) | 3304 (10.5%) | 3495 (10.3%) | 3994 (10.7%) |
| 50 to 54 years | 2556 (13.9%) | 2845 (14.1%) | 2609 (13.6%) | 3054 (14.3%) | 3186 (14.4%) | 3315 (14.6%) | 2201 (10.7%) | 3895 (14.9%) | 4027 (15%) | 4241 (14.8%) | 4457 (14.2%) | 5113 (14.8%) |
| 55 to 59 years | 2567 (16.2%) | 2817 (16.6%) | 2648 (16.3%) | 2881 (16.6%) | 3036 (17%) | 3203 (17.2%) | 3473 (14.5%) | 3643 (16.6%) | 3976 (17%) | 4355 (17%) | 4624 (16.7%) | 5171 (16.9%) |
| 60 to 64 years | 3052 (18.3%) | 3303 (18.3%) | 3092 (18.3%) | 3252 (18.2%) | 3456 (19%) | 3527 (18.7%) | 3402 (16.8%) | 3940 (18.6%) | 4137 (18.4%) | 4481 (18.7%) | 4868 (18.5%) | 5416 (18.8%) |
| >65 years | 12439 (21.8%) | 13822 (22.4%) | 13815 (23%) | 14609 (23%) | 15572 (23.6%) | 16043 (23.3%) | 20919 (22.5%) | 19316 (23.9%) | 21686 (24.7%) | 23408 (25.2%) | 24482 (24.3%) | 27352 (24.9%) |

|  | 2009 | 2010 | | 2011 | | 2012 | | 2013 | | 2014 | | 2015 | | 2016 | | 2018 | |  |
| --- | --- | --- | --- | --- | --- | --- | --- | --- | --- | --- | --- | --- | --- | --- | --- | --- | --- | --- |
| 0 to 4 years | 29360 (27.6%) | | 33087 (28.2%) | | 30485 (26%) | | 29530 (25.4%) | | 31122 (26.2%) | | 30100 (24.2%) | | 28609 (24.4%) | | 31922 (26.7%) | | 35460 (26.4%) | |
| 5 to 9 years | 13743 (25.7%) | | 15004 (26.6%) | | 13637 (24.4%) | | 14613 (24.9%) | | 14609 (24.6%) | | 14306 (24.2%) | | 14209 (23.8%) | | 15929 (26.4%) | | 8473 (20.7%) | |
| 10 to 14 years | 6272 (15.6%) | | 6873 (15.9%) | | 6219 (14.4%) | | 6771 (14.4%) | | 6957 (14.6%) | | 6633 (13.7%) | | 6276 (13.9%) | | 6640 (15.3%) | | 3536 (10%) | |
| 15 to 19 years | 3143 (3%) | | 3018 (2.8%) | | 3049 (2.7%) | | 3193 (2.8%) | | 3188 (2.8%) | | 2855 (2.5%) | | 2725 (2.5%) | | 2718 (2.8%) | | 2555 (2.6%) | |
| 20 to 24 years | 3490 (2.5%) | | 3312 (2.4%) | | 3310 (2.3%) | | 3436 (2.4%) | | 3520 (2.5%) | | 3133 (2.3%) | | 2965 (2.2%) | | 2886 (2.3%) | | 2677 (2.1%) | |
| 25 to 29 years | 3512 (2.9%) | | 3408 (2.7%) | | 3370 (2.6%) | | 3429 (2.6%) | | 3617 (2.8%) | | 3514 (2.7%) | | 3201 (2.6%) | | 2987 (2.5%) | | 2882 (2.3%) | |
| 30 to 34 years | 3297 (3.5%) | | 3311 (3.3%) | | 3326 (3.2%) | | 3352 (3.1%) | | 3617 (3.3%) | | 3342 (3.1%) | | 3153 (3%) | | 3235 (3.1%) | | 3073 (2.8%) | |
| 35 to 39 years | 3228 (4.8%) | | 3075 (4.4%) | | 3073 (4.2%) | | 3272 (4.3%) | | 3494 (4.4%) | | 3338 (4.1%) | | 3293 (4.1%) | | 3373 (4.3%) | | 3273 (3.9%) | |
| 40 to 44 years | 3606 (7.5%) | | 3590 (7.1%) | | 3645 (6.9%) | | 3620 (6.7%) | | 3725 (6.5%) | | 3623 (6.3%) | | 3477 (6.3%) | | 3418 (6.5%) | | 3398 (6%) | |
| 45 to 49 years | 4470 (11.2%) | | 4361 (10.2%) | | 4418 (9.9%) | | 4460 (9.8%) | | 4638 (9.7%) | | 4417 (9.2%) | | 4228 (9.2%) | | 4169 (9.6%) | | 4007 (8.8%) | |
| 50 to 54 years | 5451 (15.1%) | | 5325 (14%) | | 5614 (13.9%) | | 5721 (13.6%) | | 5753 (12.9%) | | 5691 (12.5%) | | 5454 (12.4%) | | 5313 (12.5%) | | 5132 (11.7%) | |
| 55 to 59 years | 5630 (17.3%) | | 5836 (16.4%) | | 6199 (16.3%) | | 6293 (16%) | | 6428 (15.2%) | | 6172 (14.3%) | | 6110 (14.4%) | | 6071 (14.7%) | | 6483 (14.6%) | |
| 60 to 64 years | 5799 (18.6%) | | 6141 (18%) | | 6533 (17.9%) | | 6598 (17.5%) | | 6756 (16.8%) | | 6836 (16.1%) | | 6617 (15.5%) | | 6546 (15.9%) | | 6614 (14.9%) | |
| >65 years | 28290 (24.3%) | | 30927 (23.9%) | | 33476 (24.4%) | | 34773 (24.2%) | | 34727 (22.7%) | | 35830 (23%) | | 35207 (22.5%) | | 35605 (22.9%) | | 38966 (22.5%) | |

Table 2S. Total absolute and relative number of ACSC hospital admissions per age groups, 2017 (2017 register included another type of age distribution)

| 2017 | |  |
| --- | --- | --- |
| 0 to 4 years | 36250 (26.1%) | |
| 5 to 9 years | 8945 (20.9%) | |
| 10 to 14 years | 3747 (10.1%) | |
| 15 to 19 years | 2721 (2.7%) | |
| 20 to 24 years | 2949 (2.3%) | |
| 25 to 34 years | 6152 (2.7%) | |
| 35 to 44 years | 6435 (4.8%) | |
| 45 to 54 years | 9150 (10.6%) | |
| 55 to 64 years | 12528 (14.9%) | |
| 65 years and more | 38141 (23.3%) | |
